# Stimulation from Cochlear Implant Electrodes Assists with Recovery from Asymmetric Perceptual Tilt: Evidence from the Subjective Visual Vertical Test

**DOI:** 10.3389/fnint.2016.00032

**Published:** 2016-09-13

**Authors:** Joshua J. Gnanasegaram, William J. Parkes, Sharon L. Cushing, Carmen L. McKnight, Blake C. Papsin, Karen A. Gordon

**Affiliations:** ^1^Archie’s Cochlear Implant Laboratory, Department of Otolaryngology, The Hospital for Sick ChildrenToronto, ON, Canada; ^2^The Institute of Medical Science, University of TorontoToronto, ON, Canada; ^3^Department of Otolaryngology—Head and Neck Surgery, University of TorontoToronto, ON, Canada

**Keywords:** vestibular, subjective visual vertical, cochlear implant, pediatric, electric stimulation, otolith, spatial perception

## Abstract

Vestibular end organ impairment is highly prevalent in children who have sensorineural hearing loss (SNHL) rehabilitated with cochlear implants (CIs). As a result, spatial perception is likely to be impacted in this population. Of particular interest is the perception of visual vertical because it reflects a perceptual tilt in the roll axis and is sensitive to an imbalance in otolith function. The objectives of the present study were thus to identify abnormalities in perception of the vertical plane in children with SNHL and determine whether such abnormalities could be resolved with stimulation from the CI. Participants included 53 children (15.2 ± 4.0 years of age) with SNHL and vestibular loss, confirmed with vestibular evoked myogenic potential (VEMP) testing. Testing protocol was validated in a sample of nine young adults with normal hearing (28.8 ± 7.7 years). Perception of visual vertical was assessed using the static Subjective Visual Vertical (SVV) test performed with and without stimulation in the participants with cochleovestibular loss. Trains of electrical pulses were delivered by an electrode in the left and/or right ear. Asymmetric spatial orientation deficits were found in nearly half of the participants with CIs (24/53 [45%]). The abnormal perception in this cohort was exacerbated by visual tilts in the direction of their deficit. Electric pulse trains delivered using the CI shifted this abnormal perception towards center (i.e., normal; *p* = 0.007). Importantly, this benefit was realized regardless of which ear was stimulated. These results suggest a role for CI stimulation beyond the auditory system, in particular, for improving vestibular/balance function.

## Introduction

Vestibular dysfunction is overwhelmingly prevalent in children with sensorineural hearing loss (SNHL; O’Reilly et al., 2010), including 40–50% of those who receive cochlear implants (CIs; Jin et al., 2006; Cushing et al., 2013; Inoue et al., 2013; Janky and Givens, 2015; Thierry et al., 2015). Moreover, the degree of hearing loss is significantly correlated with decreasing otolith function, assessed by cervical vestibular evoked myogenic potentials (cVEMPs) and the subjective visual horizontal (Tribukait et al., 2004). Such deficits are not without functional consequences, as children with concurrent vestibular and cochlear deficits perform poorly on tests of postural control (Licameli et al., 2009) and are at increased risk of CI hard failure (Wolter et al., 2015). Though Wolter et al. (2015) could not establish causality, they surmised that concurrent vestibular dysfunction can lead to an increased number of falls and associated head trauma, thereby putting the device at risk. Spatial orientation, particularly perception of the gravitational vertical, is also likely to be negatively impacted in this population due to its dependence on bilateral otolith input. On the other hand, improvements in static and dynamic equilibrium (Cushing et al., 2008) and postural stability (Eisenberg et al., 1982; Buchman et al., 2004) have been demonstrated following CI activation, perhaps due to spread of electric current from the device to the peripheral vestibular system (Parkes et al., 2016). The present study thus used the Subjective Visual Vertical (SVV) test to determine: (1) how children with SNHL using CIs perceive the vertical plane; and (2) whether or not stimulation from the CI improves the accuracy of vertical estimation.

By assessing perceived orientation of the vertical plane, the SVV test effectively evaluates for a discrepancy between the utricular input arising from each ear (Friedmann, 1971; Dieterich and Brandt, 1993; Schonfeld et al., 2010; Funabashi et al., 2015). Acute, unilateral peripheral vestibular hypofunction is commonly characterized by, among other symptoms, the presence of a perceptual tilt towards the compromised ear. This tilt typically resolves over time as central multisensory vestibular networks compensate for the asymmetric utricular input (Vibert et al., 1996, 1999; Strupp et al., 1998; Min et al., 2007; Kim et al., 2008). Transient abnormalities in the SVV have also been reported in adults with sudden unilateral SNHL (Ogawa et al., 2012). Patients with bilateral vestibular loss score similarly to control groups (Tabak et al., 1997; Guerraz et al., 2001), although a closer examination of their SVV reveals greater inter-trial variability (Funabashi et al., 2012). Much of the research to date has focused on visual vertical perception in adults, yet Brodsky et al. (2016) have recently demonstrated that the evaluation of SVV in pediatric patients is, in fact, feasible. Furthermore, the use of a smartphone application increases participant compliance without sacrificing the sensitivity of the test to peripheral vestibular loss (Brodsky et al., 2015).

Subsequent to reports on the impact of galvanic current on postural tilt (Day et al., 1997), there has been interest regarding the influence of such stimulation on spatial perception. Previous studies in animals have suggested that an anode electrode reduces vestibular afferent firing while cathodal stimulation tends to increase afferent firing rates (Goldberg et al., 1982; Kim and Curthoys, 2004). When an anode was placed over the left mastoid process of healthy participants, simulating the conditions of a unilateral peripheral vestibular lesion, these individuals’ estimations of the visual vertical were biased towards the left. Likewise, when the anode was situated over the right mastoid process, healthy participants’ SVV deviated to the right (Tardy-Gervet and Séverac-Cauguil, 1998; Volkening et al., 2014). The induced perceptual tilt of healthy participants towards the anode is comparable to that of patients with peripheral vestibular impairment, who as aforementioned often show a perceptual tilt toward their lesioned side. Although the introduction of galvanic stimulation to healthy individuals causes an abnormal shift in perception, the opposite occurs in those with a pre-existing visual tilt. Patients with right hemispheric cortical lesions demonstrate abnormal perceptual tilts to the left during baseline testing. The application of a galvanic stimulus, however, reduces perceptual tilt error, particularly when the stimulation is provided to the left vestibular end organs (Saj et al., 2006; Oppenländer et al., 2015). Given that electric current from a CI can spread outside the cochlea to adjacent structures (Cushing et al., 2006; Parkes et al., 2016), an investigation into the impact of an extrinsic source of input (i.e., stimulation from a CI) on the way children with SNHL perceive the visual vertical is warranted. We hypothesized that a large proportion of children with SNHL would demonstrate abnormalities in SVV perception and that stimulation from their CI would help rectify these impairments.

## Materials and Methods

This study was approved by an independent research ethics board (study No. 7266). Written consent was obtained from all subjects (or from parents/guardians on their behalf) prior to participation in the study.

### Participants

Review of a pre-existing database identified children and young adults with SNHL using CIs followed by our center. Participant sampling criteria included any child or young adult with SNHL and CI(s) who was willing and able to follow simple instructions during testing. Young children and individuals with developmental challenges were excluded due to anticipated difficulties with SVV assessment in those groups. Of the individuals contacted, 53 (31 males: 22 females) agreed to participate in the study. They were 15.2 ± 4.0 (mean ± 1SD; range: 7.9–27.0) years of age at the time of testing and had 10.2 ± 4.3 years of experience with their implants. Ten participants were unilaterally implanted on the right and used either a hearing aid or no device for their left ear. These children were implanted at age 7.8 ± 4.1. Of the 43 bilaterally implanted users, 6 received both implants in the same surgery at age 8.8 ± 4.2. The other 37 subjects received their first implant at 3.6 ± 2.9 years and their second at 9.7 ± 3.8 years. One child had recently been reimplanted on the left following a device failure, so this ear was not tested. The total number of ears tested was 95. The etiology of deafness varied, as outlined in Table 1. The 13 children with cochleovestibular malformations were further subdivided into four groups according to the type of anomaly: incomplete partition (IP) type II, (*n* = 8, one of whom had genetic confirmation of Pendred Syndrome); hypoplastic cochlea (*n* = 1); dilated vestibular aqueduct (*n* = 3); posterior semicircular canal (SCC) dysplasia as part of the phenotype of Waardenburg Syndrome (*n* = 1).

**Table 1:** **Etiology of hearing loss in study participants**.

Etiology of SNHL	No. (%) of participants
Cochleovestibular anomalies	13 (25)
IP type II	8 (62)
*Pendred Syndrome*	1 (13)
Hypoplastic cochlea	1 (8)
EVA	3 (23)
Posterior SCC dysplasia	1 (8)
Connexin 26 mutation	10 (19)
Usher syndrome	6 (11)
Congenital CMV infection	6 (11)
Meningitis	5 (9)
ANSD	2 (4)
Noonan syndrome	1 (2)
Unknown	10 (19)
**Total**	**53**

*IP, Incomplete Partition; EVA, Enlarged vestibular aqueduct; SCC, Semicircular canal; CMV, Cytomegalovirus; ANSD, Auditory neuropathy spectrum disorder*.

### Subjective Visual Vertical Testing

Static SVV was measured using the Visual Vertical^TM^ (Clear Health Media, Wonga Park, VIC, Australia) application on an iPod (Apple, Cupertino, CA, USA) fastened to the bottom of a bucket, a technique previously shown to reliably evaluate perceptual tilt (Zwergal et al., 2009; Brodsky et al., 2015). Testing was done in the dark, and the bucket completely filled the field of view, eliminating external visual cues. Participants sat upright with a neutral head position, and one of the examiners continuously observed the participant for the duration of the test to ensure that the head was not tilted. An external head support system was not used in an effort to control for confounding somatosensory cues. The bucket was rotated such that the red line presented by the application was oriented to the left (counter-clockwise; hereafter referred to as “left trials”) or right (clockwise; “right trials”) of the true vertical. The children were then instructed to rotate the bucket until the linear marker was congruent with their perception of vertical. After 10 s, the application calculated the difference between true and perceived vertical with an accuracy of 0.1°, representing rightward and leftward deviations with positive and negative values, respectively. A few practice trials (1–3, as needed) were conducted to ensure participants’ familiarity with the protocol, after which SVV measurements were recorded while subjects had their implants off or received unilateral electric stimulation. Six trials per condition were completed in a random order, with the bucket initially oriented to the left or right of true vertical for an equal number of trials. Testing protocol was validated in a group of adults in our laboratory with normal hearing and otolith function (*n* = 9; 6 females; 28.8 ± 7.7 [range: 19–41] years of age).

### Electric Stimuli

Electric stimulation was delivered directly to participants’ implants using Custom Sound EP^TM^software (Cochlear Corporation, Sydney, NSW, Australia) and a Nucleus Freedom processor (Cochlear Corporation, Sydney, NSW, Australia). Prior to commencing SVV testing, maximally tolerable intensity levels were determined for both 57 μs biphasic single pulses (25 μs/phase with a 7 μs interphase gap) and trains of these pulses delivered at 900 pulses/s for 4 ms at a rate of 5.1 Hz delivered from the basal (Electrode 3) or apical (Electrode 20) end of the implant array. The stimulus parameter and electrode location for SVV stimulation were determined based on which combination evoked a vestibular myogenic potential (as described in Parkes et al., 2016). When an electrically-evoked potential was not obtained, the parameter with the highest tolerable intensity was selected in order to provide the best opportunity for extra-cochlear spread of current.

### Otolith Testing

Saccular and utricular function were assessed using cervical (c) and ocular (o) VEMPs, respectively. Electromyographic potentials were evoked with a 4 ms, 500 Hz, Blackman-windowed tone burst presented at a rate of 5.1 Hz for 20 s via insert earphones to individual ears. Otolith testing was repeated with the electric stimuli described above. Using a two-channel surface electrode montage over the sternocleidomastoid and inferior oblique muscles, electromyograms were bandpass filtered (1–3000 Hz), amplified and recorded. Electromyographic activity was monitored in real time to ensure that sufficient muscle contraction was sustained. A present cVEMP response was defined as a reproducible, biphasic waveform with a peak-to-peak amplitude >20 μV. A present oVEMP response was similarly defined as a reproducible, biphasic waveform with an initial negative peak (N1) followed by a positive peak (P1; as described in Parkes et al., 2016). Acceptable amplitude peak latency ranges were defined by a normative data in children with SNHL (Xu et al., 2015).

### Data Analysis

When compared to the arithmetic mean of SVV trials, the mean of absolute SVV values more accurately reflects the perceptual abnormalities of patients with bilateral vestibular dysfunction (Funabashi et al., 2012). We expected to find abnormalities in SVV given that vestibular—specifically otolithic—dysfunction is common in children with CIs (Cushing et al., 2013). The numeric component of a participant’s SVV score in each condition was thus calculated by averaging the absolute values of the six trials. To allow for direction-specific analyses, however, the calculated SVV score retained the arithmetic sign (positive or negative) of the sum of the trials. The normal range of deviation was set to be 2° in either direction of the true vertical, based on normative data in children (Brodsky et al., 2015, 2016) and adults (Zwergal et al., 2009). The root-mean-square error (RMSE) was calculated for each participant as an indicator of spatial orientation precision. To calculate this value, the squares of the differences between the SVV measured at each trial and the overall calculated SVV score were summed and then divided by the number of trials (i.e., six). The square root of this number yielded the RMSE. This calculation can be summarized using the following formula:

(1) $$\text{RMSE}\;=\;\sqrt{\frac{\sum_{n\;=\;1}^{6}{\left({\text{SVV}}_{\text{n}}-{\text{SVV}}_{\text{calculated}}\right)}^{2}}{6}},$$

where *n* is the trial number.

Repeated Measures ANOVAs were used to determine the effect of trial and initial orientation of the linear marker on SVV score and to assess the effects of electric stimulation on perception in bilaterally implanted participants. The latter analysis excluded one reimplanted and 10 unilaterally implanted participants, as they did not receive stimulation on their left side. Bonferroni-corrected *t*-tests were used to compare subgroups *post hoc*. A Multiple Linear Regression model was also used to identify predictors of all participants’ SVV score change while receiving stimulation. Statistical analyses were conducted using RStudio Version 0.98 (R Foundation for Statistical Computing, Vienna, Austria) and SPSS Statistics 23 (IBM Corporation, Armonk, NY, USA).

## Results

**Figure 1:**
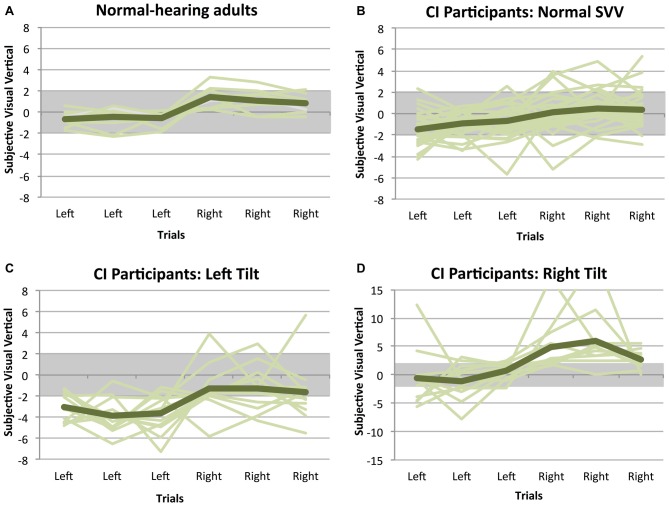
**Subjective visual vertical (SVV) measurements while participants had their implants turned off.** Positive scores indicate a perceptual tilt to the right, while negative scores represent a tilt to the left. Each line illustrates a participant’s SVV across the six trials, and the general trend for each group is shown with the darker line. In all of the normal-hearing adults **(A)**, the SVV remained within the normal range of deviation, represented with the gray bar. The same is not true of children with cochlear implants (CIs), of whom only 55% had a normal SVV score **(B)**. In the remaining group with an abnormal SVV, 54% showed a perceptual tilt to the left **(C)** and 46% demonstrated a rightward tilt **(D)**.

SVV measurements from the participants with SNHL using CIs are shown in Figure 1, along with data from normal-hearing adults for reference. In the group of adults with normal otolith function, all individuals achieved an SVV score within the normal range of deviation (i.e., perceptual tilt <2° to the left or right of zero; 0.5 ± 0.9° [mean ± SD]). In the absence of stimulation (CI processor off), only 29 children who use CIs (55%) had a normal SVV score (−0.4 ± 1.3°); the remaining 24 (45%) had a score outside the normal range of deviation. In the latter group of 24, 13 individuals (54%) showed a perceptual tilt to the left (−2.9 ± 0.4°) and 11 (46%) demonstrated a rightward tilt (3.6 ± 1.2°).

### CI Users have Contextual Visual Deficits in the Absence of Stimulation

**Figure 2:**
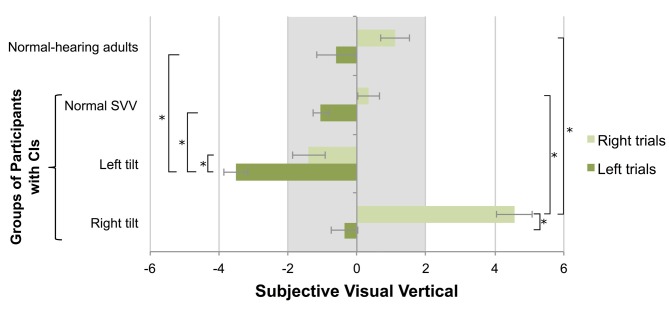
**The initial orientation of the SVV linear marker influenced participants’ estimation of the visual vertical, with normal-hearing adults’ scores shown for reference.** Positive values represent perceptual tilts to the right, and negative values indicate deviations to the left. The gray bar marks the normal range of deviation. In participants with CIs demonstrating a left perceptual tilt, exaggerated deviations to the left were observed on the left-oriented trials (mean ± SE). Conversely, participants with a rightward tilt demonstrated exaggerated deviations to the right on right-oriented trials. The asterisk represents a statistical difference with *p* < 0.05.

While the trial number was not a significant predictor of SVV measurement variability (*F*_(2,116)_ = 0.364, *p* = 0.696), the perception of the visual vertical in the absence of stimulation was biased towards the initial direction of the linear marker (*F*_(1,58)_ = 59.781, *p* < 0.001). As shown in Figure 2, there was a significant effect of direction on initial tilt (*F*_(3,58)_ = 6.253, *p* = 0.001): those children with an abnormal perceptual tilt to the left demonstrated an exaggerated deviation when the linear marker was initially oriented to the left (−3.5 ± 0.8°) compared to the right (−1.4 ± 1.8°; paired *t*-test, *p* = 0.002), and individuals with an abnormal rightward perceptual tilt showed an exaggerated deviation when the linear marker was initially oriented to the right (4.6 ± 2.8°) compared to the left (−0.3 ± 2.1°; paired *t*-test, *p* < 0.001). Furthermore, abnormalities relative to the CI group with a normal SVV score (Left trials: −1.0 ± 1.1°; Right trials: 0.3 ± 1.3°) were found in the children with left (independent *t*-tests; Left trials: *p* < 0.001; Right trials: *p* = 0.03) and right tilts (independent *t*-tests; Right trials: *p* < 0.001; Left trials: *p* = 0.36). Adult data, obtained to validate the SVV test technique, were compared to results from children with SNHL who use CIs. Abnormalities relative to this control group were indeed noted for both initial tilt directions in children with left (independent *t*-tests; Left trials: *p* < 0.001; Right trials: *p* = 0.01) and right tilts (independent *t*-tests; Right trials: *p* < 0.001; Left trials: *p* > 0.99). There were no differences between children using CIs who achieved a normal SVV score and the control group for left (independent *t*-test; *p* > 0.99) or right (independent *t*-test; *p* > 0.99) trials.

### Electric Stimulation Shifts Perception Toward Normal

Participants did not report symptoms of vertigo, dizziness, disorientation, or sensations of movement while being stimulated. Electric stimulation was provided at a maximally tolerable intensity level for each participant. The average stimulation level, given in manufacturer-defined clinical units (CU), from the right CI was 226 ± 19 CU. The range of intensity levels across all participants was 170–255 CU. The average stimulation level from the left CI was 222 ± 19 CU (range: 170–255 CU). There was no difference between the current intensities provided from the right or left CI (paired *t*-test; *t*_(41)_ = 0.891, *p* = 0.378). The average stimulation level relative to hearing threshold was 94 ± 27 CU for the right CI and 96 ± 25 CU for the left CI.

**Figure 3:**
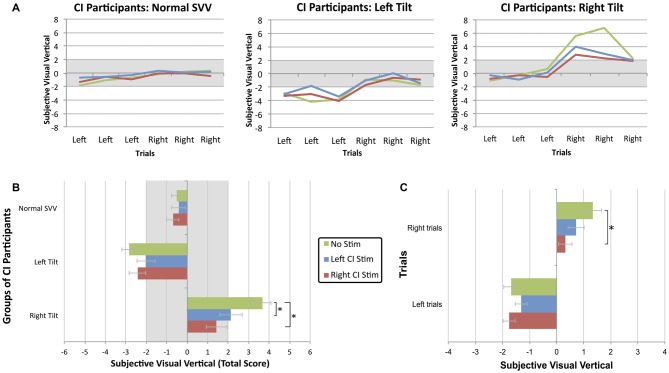
**(A)** The SVV scores across six trials of bilaterally-implanted participants demonstrate that electric stimulation from a CI shifted the perception of visual vertical toward center.** (B)** Stimulation was most beneficial when provided from the ear ipsilateral to the tilt. Each bar represents the group mean ± SE for each experimental condition. The asterisk represents a statistical difference with *p* < 0.05. **(C)** While the linear marker is known to bias the perception of visual vertical towards the direction of initial presentation, this effect was significantly reduced (mean ± SE) while participants received stimulation from their device, particularly the right CI.

As shown in Figure 3, electric stimulation from a CI shifted the perception of visual vertical toward center (*F*_(4,78)_ = 3.791, *p* = 0.007). The proportion of participants with normal scores while being stimulated from either ear improved to 74% (39/53; Fisher’s exact test, *p* = 0.068). Specifically, stimulation shifted initially abnormal SVV scores into the normal range of deviation in 14/24 participants (58%). Twenty-five of 29 (86%) participants maintained a normal SVV score while being stimulated, while the electric current resulted in abnormal tilts in four (14%). *Post hoc* analysis with Bonferroni correction further confirmed that the score of bilaterally-implanted participants with an initial normal SVV (−0.5 ± 1.3°) did not significantly differ while receiving stimulation from the left (−0.4 ± 1.5°; paired *t*-test, *p* > 0.99) or right CI (−0.7 ± 1.2°; paired *t*-test, *p* > 0.99). Individuals with a left tilt (−2.9 ± 0.4°) showed a trend toward improvement while being stimulated from their left CI (−2.0 ± 1.9°; paired *t*-test, *p* = 0.177), with no clear effects from right CI stimulation (−2.4 ± 1.5°; paired *t*-test, *p* > 0.99). Participants with a rightward tilt (3.7 ± 1.4°), in contrast, greatly benefitted from stimulation via their left CI (2.1 ± 1.0°; paired *t*-test, *p* = 0.017), and even more so with right CI stimulation (1.4 ± 1.8°; paired *t*-test, *p* = 0.002).

In bilaterally-implanted participants with either a normal or abnormal SVV score, unilateral electric current also reduced the bias of initial starting direction on estimation of visual vertical (*F*_(2,78)_ = 3.505, *p* = 0.035). As illustrated in Figure 3C, CI users were better able to compensate while receiving stimulation from their right CI (0.05 ± 1.8°; paired *t*-test, *p* = 0.034) compared to no stimulation (0.7 ± 2.9°) during trials with an initial rightward orientation. Left CI stimulation did not significantly improve perception during these right-oriented trials (0.5 ± 2.1°; paired *t*-test, *p* = 0.148). During the left-oriented trials, electric current from the right (−1.6 ± 1.8°; paired *t*-test, *p* > 0.99) or left (−1.1 ± 1.7°; paired *t*-test, *p* = 0.302) CI did not confer a significant benefit compared to no stimulation (−1.7 ± 1.7°). The linear marker’s biasing effect was not reduced to a greater extent in participants with a left- or rightward abnormal tilt compared to individuals with a normal SVV (*F*_(4,78)_ = 0.794, *p* = 0.533).

**Figure 4:**
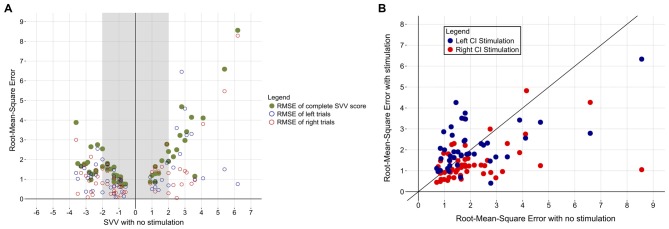
**(A)** Participants’ variability across the six trials is plotted *(green)* as a function of their baseline SVV score. Individuals with a normal SVV score *(shaded region)* were also more consistent in their responses across the six trials (i.e., low root-mean-square error [RMSE]). The RMSE of left *(blue)* and right *(red)* trials for each participant is also shown. **(B)** Participants’ RMSE decreased while they received CI stimulation, indicating increased confidence in visual vertical estimation. Red and blue dots represent RMSE while being stimulated from the right or left CI, respectively. The diagonal black line depicts no change in error in the absence vs. presence of stimulation. Data points under the line denote individuals with a lower RMSE while being stimulated, while points over the line indicate increased inter-trial variability in the presence of stimulation.

In addition to ensuring that the participants were familiar with the protocol and could confidently make their decisions in the allotted time, the within-subject variability of SVV scores was assessed to determine whether abnormal scores were marked by increased variability across trials. Figure 4A demonstrates that children who achieved a normal SVV score overall in the absence of stimulation were also more consistent across the six trials (i.e., low RMSE). On the other hand, individuals who scored abnormally on the SVV also exhibited more variability in their estimation of the visual vertical on a trial-to-trial basis. Figure 4A also shows that participants with a large perceptual tilt could be consistently abnormal (low inter-trial variability) for left and right trials or, rather, have large inter-trial variability on either side. To ensure that the observed benefit of CI stimulation was actually due to improvement in function and not the mere result of inter-trial variability, participants’ RMSE in the absence of stimulation was compared to their RMSE while being stimulated. As Figure 4B demonstrates, estimations of the visual vertical were more precise while receiving CI stimulation, as evidenced by reduced inter-trial variability. In comparison to an error in the absence of stimulation (2.0 ± 1.5°), inter-trial variability decreased while receiving right CI stimulation (1.4 ± 0.9°; paired *t*-test, *t*_(52)_ = 3.281, *p* = 0.002). There were no clear effects of left CI stimulation on inter-trial variability (2.2 ± 1.1°; paired *t*-test, *t*_(41)_ = −0.229, *p* = 0.820).

### Predictors of Perceptual Tilt and SVV Score Change

Of the 95 tested ears, acoustic cVEMPs and oVEMPs were present in 44 (46%) and 24 (25%), respectively. Electric stimuli elicited cVEMPs in 34 ears (36%) and oVEMPs in 25 (26%; Additional details of VEMP prevalence are provided in Parkes et al., 2016). Direction of initial SVV tilt was not significantly predicted by: participant age (coefficient = −0.044, *p* = 0.627), side of first implant (coefficient = 0.347, *p* = 0.509), the presence of an acoustic cVEMP (coefficient = −0.304, *p* = 0.71) or the presence of an acoustic oVEMP (coefficient = −1.631, *p* = 0.053; Multiple linear regression; *F*_(4,48)_ = 1.865, *p* = 0.132, adjusted *R*^2^ = 0.062). There was no significant difference between the absolute degree of SVV tilt in participants with (median = 1.7, 95% Confidence Interval = 1.3–2.7) and without (median = 2.0, 95% Confidence Interval = 1.3–2.7) oVEMPs (Mann-Whitney-Wilcoxon, *W* = 300.5, *p* = 0.594).

The proportions of participants with a normal or abnormal SVV score in the absence of stimulation are given in Table 2 according to etiology of hearing loss. Most children with a Connexin 26 mutation (70%) or cochleovestibular malformation (62%) were able to accurately estimate the visual vertical. Individuals with SNHL secondary to Usher Syndrome (83%) or meningitis (80%), on the other hand, exhibited perceptual deficits, with a majority of that subgroup demonstrating a rightward perceptual tilt (80% and 75%, respectively).

**Table 2:** **Proportions of participants with a normal or abnormal SVV in the absence of stimulation are described for each etiologic group**.

Etiology of SNHL	Normal SVV	Abnormal SVV	Left tilt	Right tilt
Cochleovestibular anomalies	8 (62)	5 (38)	4 (80)	1 (20)
*IP type 2*	*6 (75)*	*2 (25)*	*1 (50)*	*1 (50)*
*Hypoplastic cochlea*	*1 (100)*	*0 (0)*	–	–
*EVA*	*1 (33)*	*2 (67)*	*2 (100)*	*0 (0)*
*Posterior SCC dysplasia*	*0 (0)*	*1 (100)*	*1 (100)*	*0 (0)*
Connexin 26 mutation	7 (70)	3 (30)	2 (67)	1 (33)
Usher syndrome	1 (17)	5 (83)	1 (20)	4 (80)
Congenital CMV infection	3 (50)	3 (50)	2 (67)	1 (33)
Meningitis	1 (20)	4 (80)	1 (25)	3 (75)
ANSD	0 (0)	2 (100)	1 (50)	1 (50)
Noonan syndrome	1 (100)	0 (0)	–	–
Unknown	8 (80)	2 (20)	2 (100)	0 (0)

*Individuals with an abnormal SVV are further subdivided according to the direction of perceptual tilt. IP, Incomplete Partition; EVA, Enlarged vestibular aqueduct; SCC, Semicircular canal; SVV, subjective visual vertical; CMV, Cytomegalovirus; ANSD, Auditory neuropathy spectrum disorder*.

**Figure 5:**
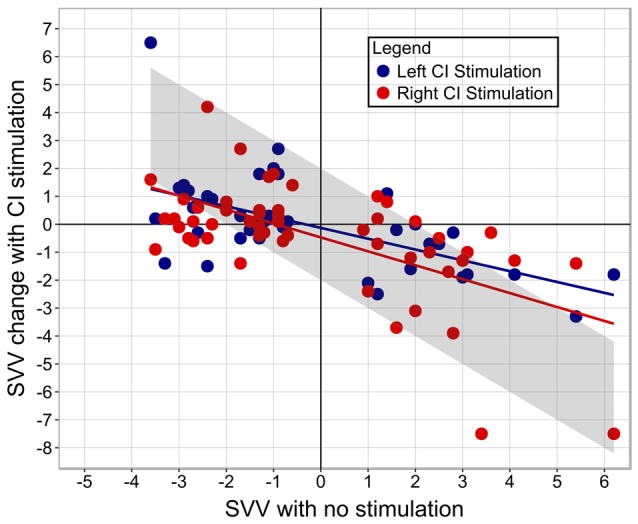
**The amount of perceptual abnormality predicts the degree of SVV score change.** The upper left quadrant illustrates that participants with a left perceptual tilt demonstrated a rightward change in SVV score with CI stimulation. The lower right quadrant illustrates that participants with a right perceptual tilt demonstrated a leftward shift in SVV score with CI stimulation. Bilaterally-implanted participants are represented by two dots, depicting SVV score change while being stimulated from their right and left CI. Unilaterally-implanted individuals are represented by a red dot due to stimulation from the right side alone. The gray bar marks the amount of change from baseline SVV (no stimulation) needed to achieve a normal SVV score.

Figure 5 demonstrates that the degree and direction initial tilt from center significantly predicted the degree and direction of the shift produced with right CI stimulation (coefficient = −0.47, *p* < 0.001). There were no clear effects of stimulation intensity normalized to hearing thresholds (coefficient = 0.002, *p* = 0.84), electric VEMP (coefficient = −0.32, *p* = 0.52), or stimulation from the ear implanted first (coefficient = 0.12, *p* = 0.74) on SVV score change (*F*_(4,46)_ = 6.818, *p* < 0.001). The degree and direction of initial tilt similarly predicted the degree and direction of SVV produced with the left CI (coefficient = −0.42, *p* < 0.001), with no clear effects of normalized stimulation intensity (coefficient = 0.01, *p* = 0.29), electric VEMP (coefficient = −0.44, *p* = 0.34) or stimulation from the ear implanted first (coefficient = 0.26, *p* = 0.4) on SVV score change (*F*_(4,35)_ = 5.912, *p* < 0.001).

## Discussion

The present study used the SVV test to investigate how children with SNHL rehabilitated with CIs perceive the vertical plane and to determine if CI stimulation affects their perception of the visual vertical. We found that a large proportion of participants with SNHL who use CIs have an asymmetric weakness that is augmented by visual tilts in the direction of their abnormality. Electric current from the CI helps to correct this abnormal perception, especially when the stimulated ear is ipsilateral to the visual tilt.

### Abnormal Perception of the Visual Vertical in CI Users

Given the high prevalence of vestibular dysfunction in children with SNHL who use CIs, we expected that a large percentage of participants would demonstrate abnormal scores on the SVV test. Indeed, 24% of subjects had an abnormal leftward tilt, and 20% showed a tilt to the right. The degree of tilt in these groups (Left tilt: −2.9 ± 0.4°; Right tilt: 3.6 ± 1.2°) is comparable to SVV scores of other groups of children with peripheral vestibular loss (Brodsky et al., 2015, 2016), providing evidence of vestibular dysfunction in children with SNHL and CIs in addition to previous reports (Cushing et al., 2013; Thierry et al., 2015; Xu et al., 2015; Parkes et al., 2016). Figure 1 (“CI Participants: Normal SVV”) demonstrates that even when CI users achieved normal overall SVV scores, there was a large amount of trial-to-trial variability in their perception of vertical. A normal score in these participants, then, would not necessarily equate to normal function (Funabashi et al., 2012), but rather increased central compensation in response to reduced vestibular integrity. In children with CIs, the direction or severity of perceptual tilt could not be predicted based on participants’ age, first implanted ear, or presence of otolith function. A previous investigation into the relationship between bone-conducted oVEMPs and the Subjective Visual Horizontal test, another functional measure of utricular health, found that rates of abnormality correlated between the two assessment methods in patients with Meniere’s disease (Lin and Young, 2011). Whereas congruence between the SVV and oVEMP tests might be expected during the acute phases of vestibular impairment, improvement in SVV performance has been reported in the weeks following the onset of vestibular dysfunction (Vibert et al., 1996, 1999; Min et al., 2007), presumably due to compensatory mechanisms initiated by vestibular processing centers. Central compensation subsequent to compromised otolith function might then explain the lack of consistency noted here between abnormal oVEMP and SVV results. Consequently, in individuals with chronic vestibular loss, such as the participants in the present study, oVEMP evaluation may perhaps be more sensitive and/or specific to end organ impairment than SVV (Valko et al., 2011). It is also possible that increasing the number of trials per condition would have enhanced the sensitivity of the test to perceptual impairment. This may have, in turn, improved the congruency between oVEMP and SVV results. More SVV trials may have also helped determine a possible association between side-specific perceptual impairments and increased variability of performance during trials which began from that impaired side.

Although visual cues are removed during SVV testing to impose dependence on otolithic input, the linear marker has been shown to bias the SVV towards the direction of initial presentation (Pagarkar et al., 2008; Toupet et al., 2015). Pagarkar et al. (2008) found this effect was enhanced in participants with unilateral peripheral vestibular deficits, and supposed that this bias was equally distributed between left and right trials. The data illustrated in Figure 2 suggest, however, that deficits were truly brought out when the marker was oriented in the same direction as the perceptual deficit. The present study furthermore highlights the importance of including left and right start positions when measuring the SVV; a participant with a right perceptual tilt could theoretically have demonstrated a normal SVV if only left-oriented trials were presented, and vice versa. Because the participants maintained a neutral head position throughout testing, the perceptual asymmetry observed in nearly half of this sample is likely due to an imbalance in bilateral vestibular neural activity. This skewed ratio of tonic activity may be present at birth due to incomplete or unilateral vestibular development or may be induced as a result of iatrogenic injuries during cochlear implantation (Jacot et al., 2009). Thus, a complex interplay between (1) congenitally present and/or inadvertently created vestibular asymmetric function; and (2) inadequate central compensation for this physiologic imbalance, may compromise the ability of these children to orient themselves spatially.

The presence of a perceptual abnormality in our participants coincides with other reports of vestibular and functional deficits in children with profound SNHL. Amongst young children (20–97 months of age) who underwent vestibular assessment before cochlear implantation, those with vestibular end organ dysfunction were more likely to acquire gross motor skills, such as head control and independent walking, at a later age than those with functional systems (Inoue et al., 2013). This developmental delay can persist or even worsen with time (Rine et al., 2000). As the interchange between visual, vestibular and somatosensory modalities can facilitate the development of compensatory mechanisms for daily function, appropriately difficult balance tasks must sometimes be administered before performance deficits are observed (Cushing et al., 2008). Similarly, children with an abnormal perception of verticality may not be aware of their perceptual deficits.

### Perceptual Accuracy Improves with CI Stimulation

The participants’ ability to estimate the gravitational vertical was significantly more accurate while they received stimulation from their CI compared to no stimulation, as illustrated in Figure 3. This finding is in accordance with numerous reports of improved balance while using a CI (Eisenberg et al., 1982; Buchman et al., 2004; Cushing et al., 2008). Figure 3 (“Normal SVV”) demonstrates that when peripheral vestibular function is intact or central perceptual centers have adequately compensated for the deficit, CI stimulation does not adversely affect the perception of vertical. The data plotted in Figure 3C indicate that although participants with a normal or abnormal SVV score were biased towards the linear marker’s initial direction, this effect was reduced while electric stimulation was provided. It has been proposed that the biasing effect is due to the incorporation of the marker’s position into short-term visual memory, which then influences the estimation of vertical (Toupet et al., 2015). Children with SNHL who use CI are not immune to this effect and may, in fact, rely more heavily on visual memory during these tasks, as exemplified in participants with an abnormal perceptual tilt. The reduction of this bias while receiving stimulation suggests that electric current recalibrates the internal perception of gravitational vertical in CI users, enhancing their ability to estimate the visual vertical independently of external cues.

Our group has previously shown that even when the otoliths themselves are non-functional, the neural components of the vestibular system remain responsive to external stimuli. This was objectively demonstrated using current from a CI to elicit otolithic responses, as measured by VEMPs, from areflexic vestibular end organs (Parkes et al., 2016). In the present study then, CI stimulation may be spreading to the peripheral vestibular system and increasing the tonic activity of the vestibular nerve. This would restore balance between previously-asymmetric vestibular inputs, in turn facilitating a more accurate spatial orientation. Sadeghi et al. (2010, 2011, 2012) further revealed that after a labyrinthine insult in nonhuman primates, neurons of the vestibular nuclei compensated for sensory loss by developing a greater sensitivity to extra-vestibular input. These findings of vestibular cross-stimulation with a CI, integration of multi-modal sensory input by vestibular nuclei, and improved perception with stimulation collectively suggest that the central nervous system is able to utilize electric stimulation from a CI as a supplement to, or in lieu of, otolith input. It is alternatively plausible that the provided electric input does not possess an inherent meaning of its own, but instead works to enhance the already-present processing strategies of the vestibular neural networks. This could be explained in part by the phenomenon of stochastic resonance, by which the deliberate introduction of noise to a system can enhance signal processing (for a review see McDonnell and Abbott, 2009). Given that CI stimulation creates an auditory percept, a third possible explanation takes into account the role of auditory processing centers. Although the stimulus itself does not provide meaningful input for one specific sensory modality (auditory or vestibular), it is conceivable that vestibular, auditory and perhaps other sensory modalities work synergistically to facilitate a gestalt-like interpretation of the CI stimulation.

The data shown in Figure 3B illustrate that while electric current from either ear improved perceptual accuracy, stimulation from the side ipsilateral to the tilt conferred an even greater advantage. Improvements in vertical perception have also been observed in patients with right-hemisphere stroke who received galvanic vestibular stimulation, although these patients were more accurate with contralateral stimulation (i.e., from their left side; Saj et al., 2006; Oppenländer et al., 2015). In as much as SVV tilt manifestation is representative of lesion location in the vestibular pathway (Dieterich and Brandt, 1993; Brandt et al., 1994; Yang et al., 2014), the most beneficial site of stimulation is potentially dependent on where input is needed: peripheral vestibular dysfunction requires ipsilateral stimulation, while contralateral stimulation restores input to more rostral deficiencies.

Linear regression analysis of data shown in Figure 5 indicated a significant correlation between the degree of abnormality and the amount of SVV score change, which is perhaps why statistically significant improvements were detected in those with a right perceptual tilt, but not those with a left tilt; the group with a right deficit exhibited more change because they had more of an initial abnormality. The intensity of stimulation seemingly does not dictate the amount of gained perceptual benefit, as indicated by the linear regression. Although all participants received a comfortably loud stimulus, those who tolerated higher intensity levels did not show a greater shift in SVV score. Moreover, the aforementioned study of galvanic vestibular stimulation reported improved vertical estimation while using a subthreshold stimulus (Oppenländer et al., 2015). These findings imply that the perceptual benefit is contingent on the successful activation of the vestibular system rather than being a function of the magnitude of stimulation provided. As such, it is conceivable that the current levels used daily by children with CIs may be sufficient to cross-stimulate the vestibular system and confer a functional benefit.

This study is part of a larger endeavor to characterize CI-mediated stimulation of the vestibular system along with related effects on balance and perception. As a result, short pulses of CI stimulation were deliberately provided at comfortably loud intensities in order to maximize extra-cochlear current spread and evoke vestibular potentials. Although participants heard this stimulation, this stimulus paradigm is not used to encode complex sounds such as speech. Nonetheless, children’s balance improves with their CIs on while using normal speech processing settings (Cushing et al., 2008). Given the results presented here, it is possible that these more typical stimulation modes provide vestibular cross-stimulation. Further investigation is needed to confirm whether this is the mediator for the observed functional benefits.

In order to control for a short-term learned effect, SVV trials were randomized between the No-Stimulation and Stimulation conditions. The observed difference in SVV scores indicates that the conferred benefit lasts for the duration of stimulation and quickly decays thereafter. It is possible, though, that stimulation cues which provide meaningful input may have longer lasting effects: studies are now being conducted in our lab in which three-dimensional head movements are relayed to children through their CIs. Anecdotal reports from parents and limited data from children who have participated in these pilot studies have demonstrated enhancements in balance coping mechanisms that are long-lasting and transferrable into daily activities. As such, the duration of improvement may perhaps be a function of the type of stimuli provided (i.e., stimulus parameters) and the information encoded by this stimulation.

Comparing the amount of stimulation necessary to observe balance or perceptual improvement with that required to evoke a vestibular reflex may also clarify the relationship between electrophysiologic (e.g., VEMP) and functional (e.g., SVV) measures. In addition, the current study protocol examined the impact of unilateral electric stimulation on perceptual tilt. As the majority of children with SNHL seen at our institution receive two implants, it would be worthwhile to explore how the vestibular system responds to the simultaneous delivery of bilateral stimulation, particularly if this confers a greater benefit in comparison to unilateral input. Additionally, though the children assessed in the present study were physically able to complete the testing protocol, one can imagine the challenges associated with the use of this “bucket” technique in younger children, who have not yet developed the strength needed to support the bucket’s weight. Future studies should therefore explore the efficacy of a commercially-available goggle system for SVV evaluation (cSVV, Chronos Vision, Germany), which might then aid with extending the applicability of this test to a younger population.

## Conclusion

The current study provides evidence that a large proportion of children who have SNHL and use CIs have an abnormal perception of vertical. Furthermore, those with an abnormal tilt show a directional bias based on initial context, which suggests an asymmetric weakness and could result in an inability to compensate from visual tilts in the direction of their abnormality. The finding that electrical pulses from the CI helped to correct this perception, especially when provided from the side ipsilateral to the tilt, suggests a therapeutic benefit of the implant beyond its main auditory target.

## Author Contributions

All authors provided direct, substantial, and intellectual contributions to data collection and/or preparation and approval of the manuscript for publication.

## Funding

This research was supported by a grant from the Harry Barberian Research fund.

## Conflict of Interest Statement

BCP is a member of the speaker’s bureau for Cochlear Americas Corporation. All other authors have no conflicts of interest to declare.
